# Impact of salt and exogenous AM inoculation on indigenous microbial community structure in the rhizosphere of dioecious plant, *Populus cathayana*

**DOI:** 10.1038/s41598-021-97674-w

**Published:** 2021-09-15

**Authors:** Na Wu, Zhen Li, Ming Tang

**Affiliations:** 1 State Key Laboratory of Conservation and Utilization of Subtropical Agro-bioresources, Lingnan Guangdong Laboratory of Modern Agriculture, College of Forestry and Landscape Architecture, South China Agricultural University, Guangzhou, 510642 Guangdong China; 2 Institute of Applied Biotechnology, School of Life Science, Shanxi Datong University, Datong, 037009 Shanxi China

**Keywords:** Microbiology, Plant sciences

## Abstract

The sex-specific physical and biochemical responses in dioecious plants to abiotic stresses could result in gender imbalance, and how to ease the current situation by microorganisms is still unclear. Using native soil where poplars were grown, growth parameters, soil physicochemical properties in the rhizosphere soil of different sexes of *Populus cathayana* exposed to salt stress and exogenous arbuscular mycorrhizal (AM) inoculation were tested. Besides, the sex-specific microbial community structures in the rhizosphere soil of different sexes of *Populus cathayana* were compared under salt stress. To identify the sex-specific microbial community characteristics related to salinity and AM symbiosis, a combined qPCR and DGGE method was used to monitor microbial community diversity. Seedlings suffered severe pressure by salt stress, reflected in limited growth, biomass, and nutrient element accumulation, especially on females. Exogenous AM inoculation treatment alleviated these negative effects, especially under salt treatment of 75 mM. Compared with salt effect, exogenous AM inoculation treatment showed a greater effect on soil physical–chemical properties of both sexes. Based on DGGE results, salt stress negatively affected fungal richness but positively affected fungal Simpson diversity index, while exogenous AM inoculation treatment showed the opposite effect. Structural equation modeling (SEM) was performed to show the causal relationships between salt and exogenous AM inoculation treatments with biomass accumulation and microbial community: salt and exogenous AM inoculation treatment showed complicated effects on elementary concentrations, soil properties, which resulted in different relationship with biomass accumulation and microbial community. Salt stress had a negative effect on soil properties and microbial community structure in the rhizosphere soil of *P. cathayana*, whereas exogenous AM inoculation showed positive impacts on most of the soil physical–chemical properties and microbial community status.

## Introduction

Having an appropriate concentration of salt ions in the soil solution is very important for the completion of the normal plant life cycle; however, global periodic drought exacerbates soil salinization^1^. Soil salinity can harm plant growth by interfering with physiological and biochemical mechanisms^2^. To overcome the detrimental effects of salinity, plants adopt a variety of strategies, such as branched root systems and the development of AM symbiosis underground^3^. As an important part of the rhizosphere micro-ecosystem, by affecting the root exudation process through a huge underground hyphal network, AM symbiosis indirectly regulates microbial community diversity^4^. Kohler et al.^5^ found that AM inoculation had an obvious effect on microbial community composition in the rhizosphere soil of *Dorycnium pentaphyllum*. To test how AM fungi affected soil decomposition process, Gui et al.^6^ used a two-chamber microscopic observation and found that AM fungi inhibited microbial community development and increased the decomposition rate at the later stage of litter decomposition. AM formation can improve soil fertility and enhance plant nutrient absorption, which provides a certain theoretical basis for the use of microbial remediation technology to improve environmental quality.

Bacteria and fungi in soil have been recognized as two important microbial populations that indicate soil health and productivity^7^. These terrestrial microorganisms play a crucial role in ecological processes^8^. Microbes developed different adaptation mechanisms to resist abiotic stresses. Understanding how soil microorganisms respond to salt stress and AM symbiosis is a vital step in developing restoration strategies. Microbial community diversity is an important indicator of soil quality, so changes in microbial community composition are usually considered a sensitive indicator of soil ecological status. Exogenous AM fungi and indigenous soil microbes are interrelated and interact with each other^6^. The application of AM strain to improve the soil microbial community and whether AM fungi can be applied in the field of rhizosphere ecology still needs more evaluation.

*Populus cathayana*, a dioecious species, mainly distributed in northwestern China, exhibits fast growth and strong adaptability. Soil salinity is prevalent and one of the most serious environmental stresses in northwestern China^9^. Many studies have suggested that female *P. cathayana* were more sensitive than males when exposed to abiotic stresses^10–12^. However, few studies focus on how the microbial community structure in the rhizosphere soil of dioecious plants changes to salinity conditions and exogenous AM inoculation. With limited research on micro-organism’s variation in the rhizosphere soil of *P*. *cathayana*, we aimed to identify sex-specific responses in microbial community diversity to AM formation and salinity by nested PCR-DGGE analysis^13,14^. We examined two hypotheses in this study: i) Salt stress and exogenous AM inoculation would modify the rhizospheric environment of *P*. *cathayana* of both sexes. ii) Differences between the microbial communities from two genders to salt and AM inoculation would be detected.

## Results

### Plant parameters

Salt treatment of 75 mM limited plant growth rate of height (GRH), biomass accumulation and P content of both genders significantly (Table 1). To N content, plants those subjected to salt treatment of 75 mM accumulated more N content in the leaf of males and the root of females than those under 0 mM condition. Compared with non-exogenous AM application treatment of both sexes, the application of exogenous AM inoculation alleviated the damage caused by salt stress, mainly manifested in the growth rates, biomass accumulation and nutrient acquisition of the cuttings. Among male seedlings under 0 mM condition, plants that received exogenous AM inoculation treatment showed more biomass and P content accumulation of the root than those did not, whereas no difference was detected in growth of stem length, P content of the leaf, N content of both the root and leaf between male seedlings received exogenous AM inoculation and those did not. Under salt treatment of 75 mM, except P content of the leaf, exogenous AM inoculation enhanced male seedlings’ growth indexes observed. Among females, GRH and P content of the leaf of seedlings inoculated by exogenous AM fungi were significantly higher than those recorded for uninoculated seedlings. However, exogenous AM inoculation showed a contrary effect on N content of the leaf of females compared with on other indexes. Under salt treatment of 75 mM, females inoculated by exogenous AM fungi showed better performance in all the indexes. Irrespective of exogenous AM inoculation, the reduction in nutrient contents in females was higher than that in males, illustrating that salt stress caused more negative effects in females than in males.

**Table 1:** Effects of AMF inoculation on growth parameters and nutrient acquisition of *P. cathayana* females and males under different salt conditions.

Gender	Salt treatment (mM)	Inoculation Treatment	GRH (cm d^−1^)	Biomass (g)	N content (mg g^−1^ DW)		P content (mg kg^−1^ DW)	
					Leaf	Root	Leaf	Root
Male	0	AM	1.34 ± 0.06a	24.57 ± 1.76a	10.23 ± 0.28a	7.29 ± 0.11bc	370.34 ± 14.33a	355.77 ± 9.50a
		NM	1.32 ± 0.04a	21.66 ± 0.41b	10.38 ± 0.33a	7.13 ± 0.13c	377.99 ± 10.11a	341.73 ± 13.46b
	75	AM	0.87 ± 0.04b	15.70 ± 1.55c	9.53 ± 0.36b	7.40 ± 0.25a	323.47 ± 17.36c	306.84 ± 10.96c
		NM	0.71 ± 0.02c	12.41 ± 1.04d	7.89 ± 0.37d	6.86 ± 0.11d	322.22 ± 12.71c	288.13 ± 21.55d
	*P*_salt_		**	**	**	NS	**	**
	*P*_AM inoculation_		**	**	**	**	NS	**
	*P*_salt×AM inoculation_		**	NS	**	*	NS	NS
Female	0	AM	1.42 ± 0.05a	22.31 ± 1.9aa	8.91 ± 0.47c	8.16 ± 0.61a	338.92 ± 14.09c	288.73 ± 18.54d
		NM	1.36 ± 0.05a	22.11 ± 1.5ab	9.31 ± 0.26b	8.19 ± 0.17A	350.96 ± 11.15b	302.29 ± 12.80c
	75	AM	0.76 ± 0.04c	15.50 ± 0.62c	9.03 ± 0.28b	6.97 ± 0.14d	240.44 ± 15.19d	215.99 ± 8.77e
		NM	0.64 ± 0.03d	11.81 ± 0.51d	8.58 ± 0.34c	6.41 ± 0.33e	217.44 ± 14.95e	183.51 ± 14.14f.
	*P*_salt_		**	**	NS	**	NS	*
	*P*_AM inoculation_		**	**	*	**	**	**
	*P*_salt×AM inoculation_		NS	**	**	**	**	**
*P*_gender_			NS	*	**	**	**	**
*P*_salt×gender_			**	**	**	**	*	*
*P*_AM inoculation×gender_			**	**	**	*	**	*
*P*_salt×AM inoculation×gender_			*	**	NS	**	**	**

AM, exogenous AMF inoculation; NM, non-exogenous AMF inoculation; 0 mM, without salt stress; 75 mM, under salt stress. **: significant effect at *P* ≤ 0.01; *: significant effect at 0.01 ≤ P ≤ 0.05; NS: no significant effect *P* > 0.05. Different lowercase letters indicate significant difference (*P* ≤ 0.05), the data are means ± SD (n = 6).

Two-way ANOVA results indicated salt treatment affected GRH, biomass, N content of the leaf, and P contents of the leaf and root of male seedlings and GRH, biomass, N and P contents of the root of female seedlings significantly, while AM inoculation treatment affected all the growth indexes of both sexes (except P content of the male leaf) regardless of salt treatment significantly. Three-way ANOVA results suggested gender effect and interactions of salt treatment × gender and AM inoculation treatment × gender existed in all the growth indexes (except gender on GRH). And GRH, biomass, N content of the root and P contents of the leaf and root were significantly affected by the interaction of three factors.

### AM colonization, spore density and glomalin content

AM colonization were detected in roots of both seedlings received exogenous AM inoculation treatment and those did not. Among seedlings with exogenous AM inoculation, salt stress had significantly negative effect on AM colonization and AM spore density of plants in both genders. Among seedlings without exogenous AM inoculation, salt significantly decreased AM colonization rate of male seedlings and AM spore density of females. However, exogenous AM inoculation treatment had a similar positive effect on AM colonization and AM spore density of both genders under either 0- or 75-mM salt conditions (Table 2). Salt treatment had significantly negative effects on the total glomalin (TG) and easily extractable glomalin (EEG) contents of both sexes (except EEG content in male), which went against of exogenous AM inoculation treatment. Meanwhile, compared with male seedlings without exogenous AM inoculation, uninoculated females showed higher AM colonization rate under both salt conditions. Two-way ANOVA results suggested that, except the effect of exogenous AM inoculation treatment on AM colonization rate of females, salt treatment and exogenous AM inoculation treatment affected AM colonization rate, AM spore density and glomalin contents of both genders. Three-way ANOVA results indicated that the interaction of salt treatment × gender and AM inoculation treatment × gender affected all these parameters significantly, and the interaction of 3 factors showed significant effect on AM colonization rate, AM spore density and TG content.

**Table 2:** Effects of exogenous AM inoculation on AM colonization, AM spore density and glomain contents in the rhizosphere of *P*. *cathayana* males and females under different salt conditions.

Gender	Salt treatment (mM)	Inoculation treatment	AM colonization rate (%)	AM spore density (10 g^−11^)	TG content (g kg^−1^)	EEG content (g kg^−1^)
Male	0	AM	96.28 ± 2.38a	38.31 ± 2.39b	3.13 ± 0.230a	0.54 ± 0.08a
		NM	36.80 ± 2.08d	15.85 ± 3.88e	2.91 ± 0.07b	0.25 ± 0.07c
	75	AM	78.98 ± 3.84b	33.16 ± 2.64c	2.72 ± 0.12c	0.54 ± 0.07a
		NM	21.09 ± 3.36e	14.13 ± 3.89e	1.72 ± 0.63d	0.12 ± 0.01d
	*P*_salt_		**	*	**	**
	*P*_AM inoculation_		**	**	**	**
	*P*_salt×AM inoculation_		NS	NS	**	**
Female	0	AM	91.70 ± 5.51a	43.14 ± 3.39a	3.39 ± 0.09a	0.60 ± 0.04a
		NM	56.27 ± 6.06c	24.35 ± 4.27d	2.61 ± 0.15c	0.21 ± 0.01c
	75	AM	83.83 ± 5.71b	12.65 ± 2.01e	2.53 ± 0.27c	0.41 ± 0.04b
		NM	60.51 ± 7.90c	9.12 ± 1.37f.	1.11 ± 0.14e	0.14 ± 0.01d
	*P*_salt_		**	**	**	**
	*P*_AM inoculation_		NS	**	**	**
	*P*_salt×AM inoculation_		*	**	**	**
*P*_gender_			**	**	**	*
*P*_salt×gender_			**	*	*	**
*P*_AM inoculation×gender_			**	*	*	*
*P*_salt×AM inoculation×gender_			*	*	*	NS

AM, exogenous AM inoculation; NM, non-exogenous AM inoculation; 0 mM, without salt stress; 75 mM, under salt stress. **: significant effect at *P* ≤ 0.01; *: significant effect at 0.01 ≤ *P* ≤ 0.05; NS: no significant effect *P* > 0.05. Different lowercase letters indicate significant difference (*P* ≤ 0.05), the data are means ± SD (n = 6).

### Soil physical–chemical properties and enzyme activities

The pH value ranged around 8.05 (from 8.03 to 8.10). As showing in Table 3, salt stress significantly increased EC of both genders. Among seedlings those had not received exogenous AM inoculation treatment, the available P and available K contents of both sexes, and available N of males under 0 mM salt conditions were significantly higher than those under 75 mM salt conditions. Among females those had received exogenous AM inoculation treatment, seedlings under 0 mM showed significantly higher available N and available K contents than those under 75 mM salt conditions, whereas no difference was detected in available nutrient elements recorded for males with exogenous AM inoculation. For SOC content from rhizosphere, there was no difference among females of different treatment.

**Table 3:** Effects of exogenous AM inoculation on soil physical and chemical properties in the rhizosphere of *P*. *cathayana* males and females under different salt conditions.

Gender	Salt stress (mM)	Inoculation treatment	pH	EC (μs cm^−1^)	SOC (g kg^−1^)	Available N (mg kg^−1^)	Available P (mg kg^−1^)	Available K (mg kg^−1^)
Male	0	AM	8.06 ± 0.04	4.23 ± 0.32e	10.93 ± 0.33a	41.04 ± 1.44b	14.47 ± 1.40a	143.10 ± 19.34ab
		NM	8.05 ± 0.01	6.49 ± 0.40d	8.84 ± 0.27c	40.22 ± 0.85b	11.50 ± 0.30b	142.90 ± 4.05a
	75	AM	8.03 ± 0.06	36.19 ± 0.93a	11.04 ± 0.71a	40.31 ± 2.15b	13.58 ± 1.41a	138.42 ± 4.20b
		NM	8.04 ± 0.08	30.19 ± 1.04b	8.76 ± 0.86c	38.40 ± 0.61c	9.48 ± 0.28c	134.63 ± 13.36b
	*P*_salt_		NS	**	NS	*	**	**
	*P*_AM inoculation_		NS	*	*	*	**	NS
	*P*_salt×AM inoculation_		NS	**	NS	NS	NS	NS
Female	0	AM	8.10 ± 0.04	8.43 ± 0.31c	10.08 ± 0.71b	44.05 ± 1.97a	14.62 ± 0.83a	140.37 ± 4.91ab
		NM	8.07 ± 0.04	8.84 ± 0.26c	10.02 ± 0.41b	35.83 ± 1.03c	14.95 ± 0.55a	144.63 ± 6.80a
	75	AM	8.07 ± 0.01	31.65 ± 0.54b	10.10 ± 0.38b	36.97 ± 1.65c	14.88 ± 0.57a	133.69 ± 10.09b
		NM	8.06 ± 0.02	31.79 ± 1.22b	9.64 ± 0.53b	35.48 ± 0.23c	11.00 ± 0.71b	133.81 ± 3.71b
	*P*_salt_		NS	**	NS	**	**	NS
	*P*_AM inoculation_		NS	*	NS	**	**	**
	*P*_salt×AM inoculation_		NS	NS	NS	**	**	NS
*P*_gender_			NS	*	NS	**	**	**
*P*_salt×gender_			NS	**	NS	**	**	*
*P*_AM inoculation×gender_			NS	*	**	**	**	**
*P*_salt×AM inoculation×gender_			NS	NS	NS	**	**	NS

AM, AMF inoculation; NM, non-inoculation; 0 mM, without salt stress; 75 mM, under salt stress; M, male; F, female. Different normal letters in the same column mean 5% significant differences.

Salt stress significantly decreased the activities of urease, catalase, dehydrogenase, sucrase and ALP in the rhizosphere of *P. cathayana* of both genders (Table 4). Under either salt conditions, exogenous AM inoculation treatment significantly enhanced activities of catalase, dehydrogenase, and ALP of both sexes. In contrast, among urease and surcease activities, salt treatment showed greater effect than AM inoculation treatment.

**Table 4:** Effects of AMF inoculation on the soil enzyme activities in the rhizosphere of *P*. *cathayana* males and females under different salt conditions.

Gender	Salt treatment (mM)	Inoculation treatment	Urease (mg g^−1^ h^−1^)	Catalase (ml g^−1^ (20 min) ^−1^)	Dehydrogenase (mg g^−1^ h^−1^)	Sucrase (mg g^−1^ h^−1^)	ALP (mg g^−1^ h^−1^)
Male	0	AM	0.81 ± 0.03a	3.07 ± 0.08a	30.94 ± 4.02a	2.29 ± 0.08b	1.27 ± 0.06a
		NM	0.80 ± 0.03a	1.44 ± 0.07c	25.58 ± 3.73b	2.28 ± 0.29b	0.84 ± 0.02d
	75	AM	0.53 ± 0.02d	1.59 ± 0.07b	20.02 ± 0.67c	1.94 ± 0.10c	0.90 ± 0.02c
		NM	0.55 ± 0.02d	0.92 ± 0.03e	11.13 ± 0.62f.	1.92 ± 0.12c	0.63 ± 0.02e
	*P*_salt_		**	**	**	**	**
	*P*_AM inoculation_		NS	**	*	NS	**
	*P*_salt×AM inoculation_		NS	**	**	NS	*
Female	0	AM	0.69 ± 0.02b	3.10 ± 0.08a	28.45 ± 2.54a	2.50 ± 0.15a	1.13 ± 0.04b
		NM	0.71 ± 0.03b	1.66 ± 0.04b	23.16 ± 1.51b	2.49 ± 0.14a	0.92 ± 0.02c
	75	AM	0.58 ± 0.03c	1.33 ± 0.10d	18.40 ± 0.42d	2.06 ± 0.07c	0.62 ± 0.10e
		NM	0.57 ± 0.02c	0.84 ± 0.04e	15.05 ± 0.47e	1.95 ± 0.05c	0.48 ± 0.05f.
	*P*_salt_		NS	*	**	NS	**
	*P*_AM inoculation_		**	*	*	**	**
	*P*_salt×AM inoculation_		NS	NS	**	NS	*
*P*_gender_			**	NS	**	**	**
*P*_salt×gender_			**	**	*	*	**
*P*_AM inoculation×gender_			**	**	*	**	*
*P*_salt×AM inoculation×gender_			NS	*	*	NS	*

AM, AMF inoculation; NM, non-inoculation. Different normal letters in the same column mean 5% significant differences.

Two-way ANOVA results indicated that, except available K content from rhizosphere of female cuttings, the soil EC, available nutrient elements contents were significantly affected by salt treatment of both sexes, and exogenous AM inoculation had effect on EC, SOC, available N and available P contents of soil from males and EC, available nutrient elements of soil from females. Among soil enzymatic activities, salt treatment showed a significant effect on all 5 enzymatic activities of soil from males and only catalase, dehydrogenase, and ALP activities of soil from females. However, AM inoculation treatment affected catalase, dehydrogenase, and ALP activities of soil from males and all 5 enzymes recorded for females. Three-way ANOVA results suggested that, except gender effect on catalase activity, effects of gender and interactions of salt treatment × gender, AM inoculation treatment × gender existed in EC, available nutrient elements, and soil enzymatic activities.

### Cluster analysis based on the microbial-DGGE profile

**Figure 1:**
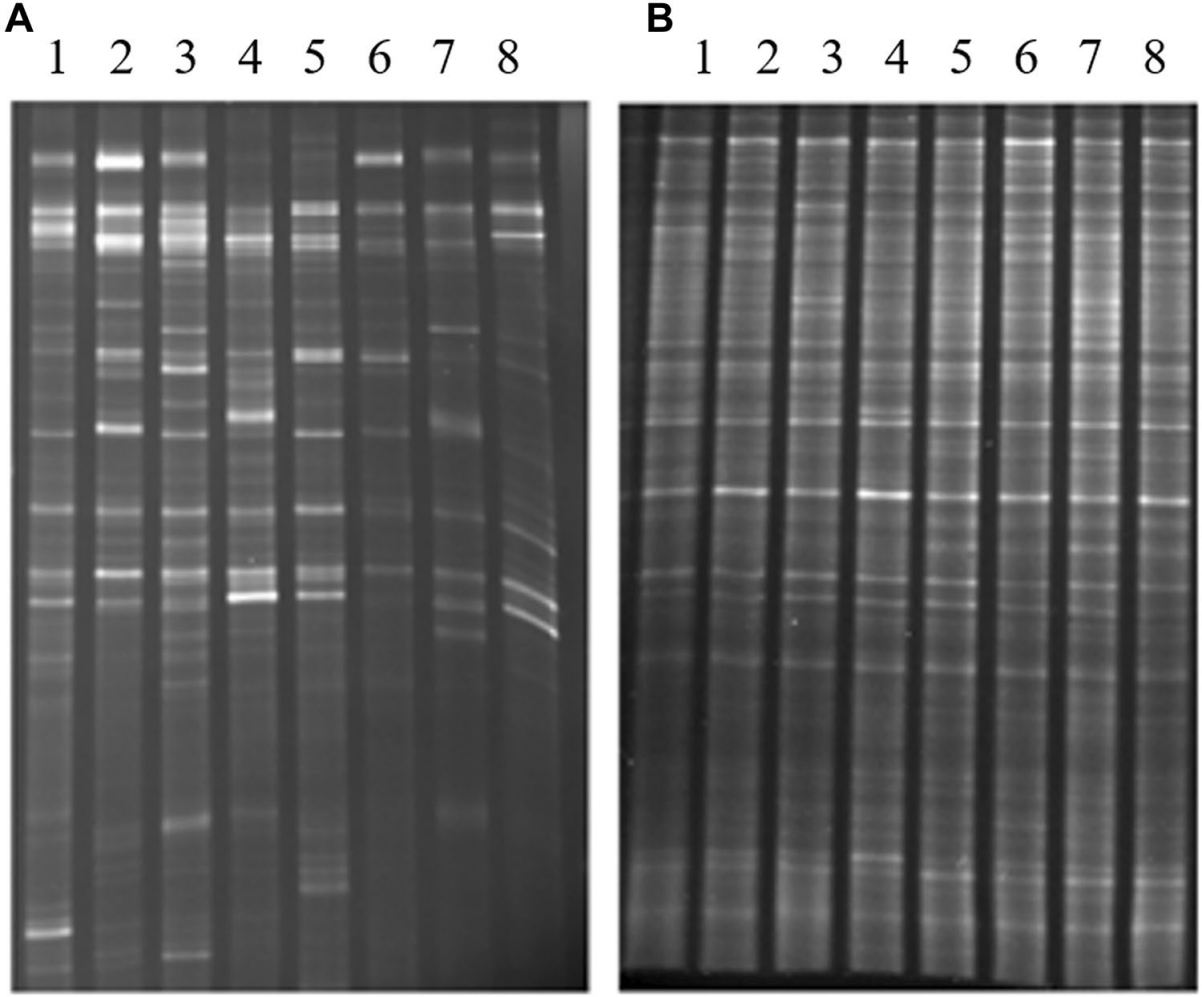
DGGE maps of fungal (**A**) and bacterial (**B**) communities. **A** DGGE map of bacterial community; **B** DGGE map of fungal community; 1: AM M 0 mM; 2: AM M 75 mM; 3: AM F 0 mM; 4: AM F 75 mM; 5: NM M 0 mM; 6: NM M 75 mM; 7: NM F 0 mM; 8: NM F 75 mM. *Note*: AM: exogenous AM inoculation; NM: non-exogenous AM inoculation; M: males; F: females; 0 mM: without salt stress; 75 mM: under salt stress.

**Figure 2:**
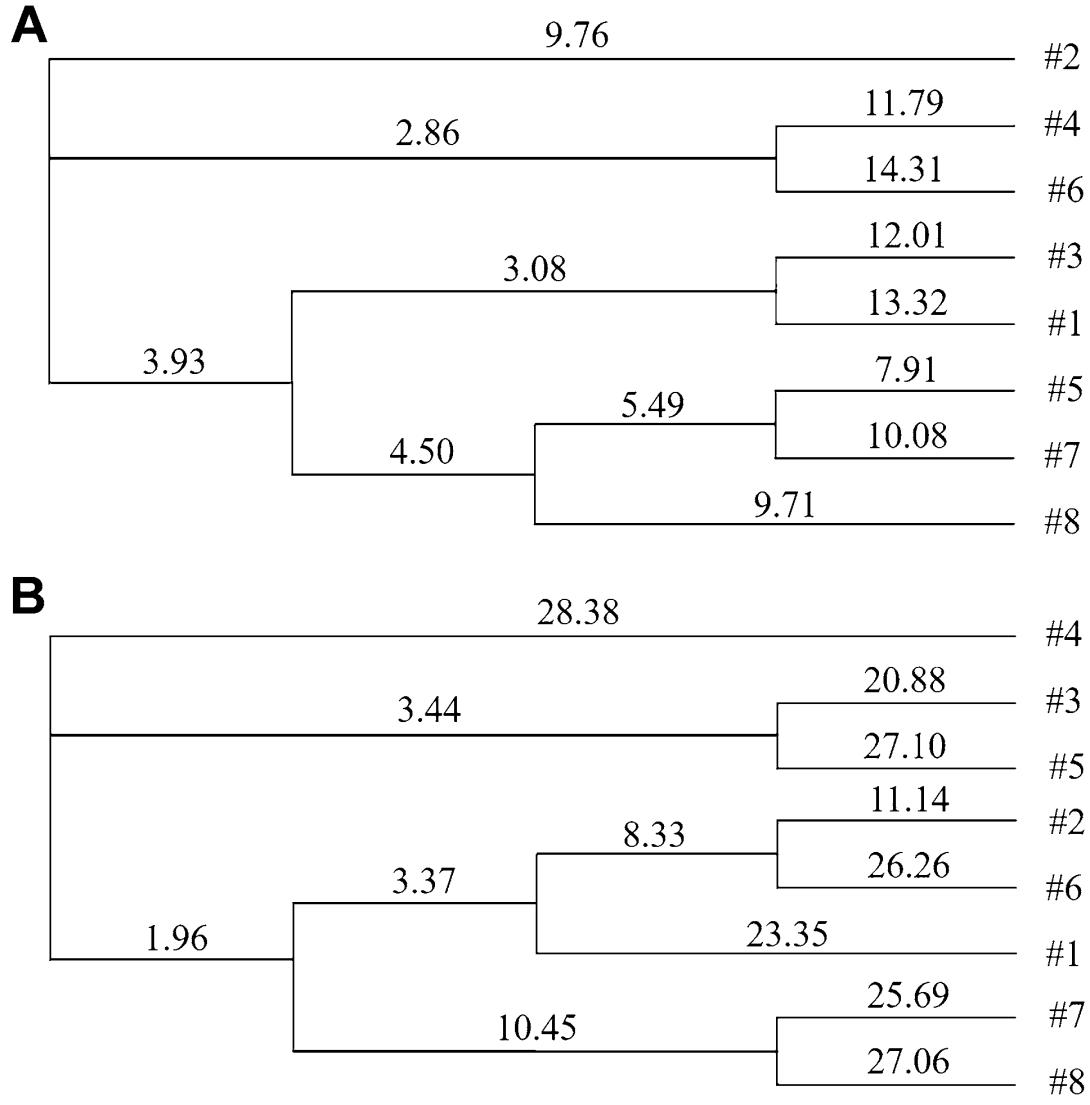
Cluster analysis generated based on bateria- (**A**) and fungi-DGGE (**B**) profile. **#**1: AM M 0 mM; #2: AM M 75 mM; #3: AM F 0 mM; #4: AM F 75 mM; #5: NM M 0 mM; #6: NM M 75 mM; #7: NM F 0 mM; #8: NM F 75 mM. *Note*: AM: exogenous AM inoculation; NM: non-exogenous AM inoculation; M: males; F: females; 0 mM: without salt stress; 75 mM: under salt stress.

Figure 1 shows that *R. irregularis* has a significant impact on the microbial community structure of *P. cathayana*. The cluster analysis of DGGE patterns showed that the 8 treatments were separated into three distinct groups. As shown in Fig. 2, there was a close relationship between the indigenous microorganisms and exogenous AM inoculation treatments without salt stress (treatments 1 and 3, treatments 5 and 7), indicating that the similarity of target bacteria in the rhizospheres of female and male cuttings was higher irrespective of exogenous AM inoculation. Under 75 mM salt stress, the indigenous microorganisms in the rhizosphere of female cuttings (treatments 4 and 8) were obviously different.

As shown in Fig. 2, there was a close relationship between the indigenous microorganisms and exogenous AM inoculation treatment of male cuttings under salt stress (treatments 2 and 6), indicating that the similarity of target fungi in the rhizosphere of male cuttings was higher regardless of exogenous AM inoculation under salt stress. Indigenous microorganisms and exogenous AM in the rhizosphere of male cuttings (treatments 1 and 5) were far apart under non-salt stress, illustrating that exogenous AM inoculation induced significant changes in the target fungi in the rhizosphere of male cuttings. In addition, salt stress, and the non-salt stress treatments of female cuttings (treatments 7 and 8) with indigenous microorganisms was far apart, illustrating that salt stress did not cause significant changes in the target fungal community in the rhizosphere of female cuttings.

### Microbial community structure based on DGGE profiles

**Figure 3:**
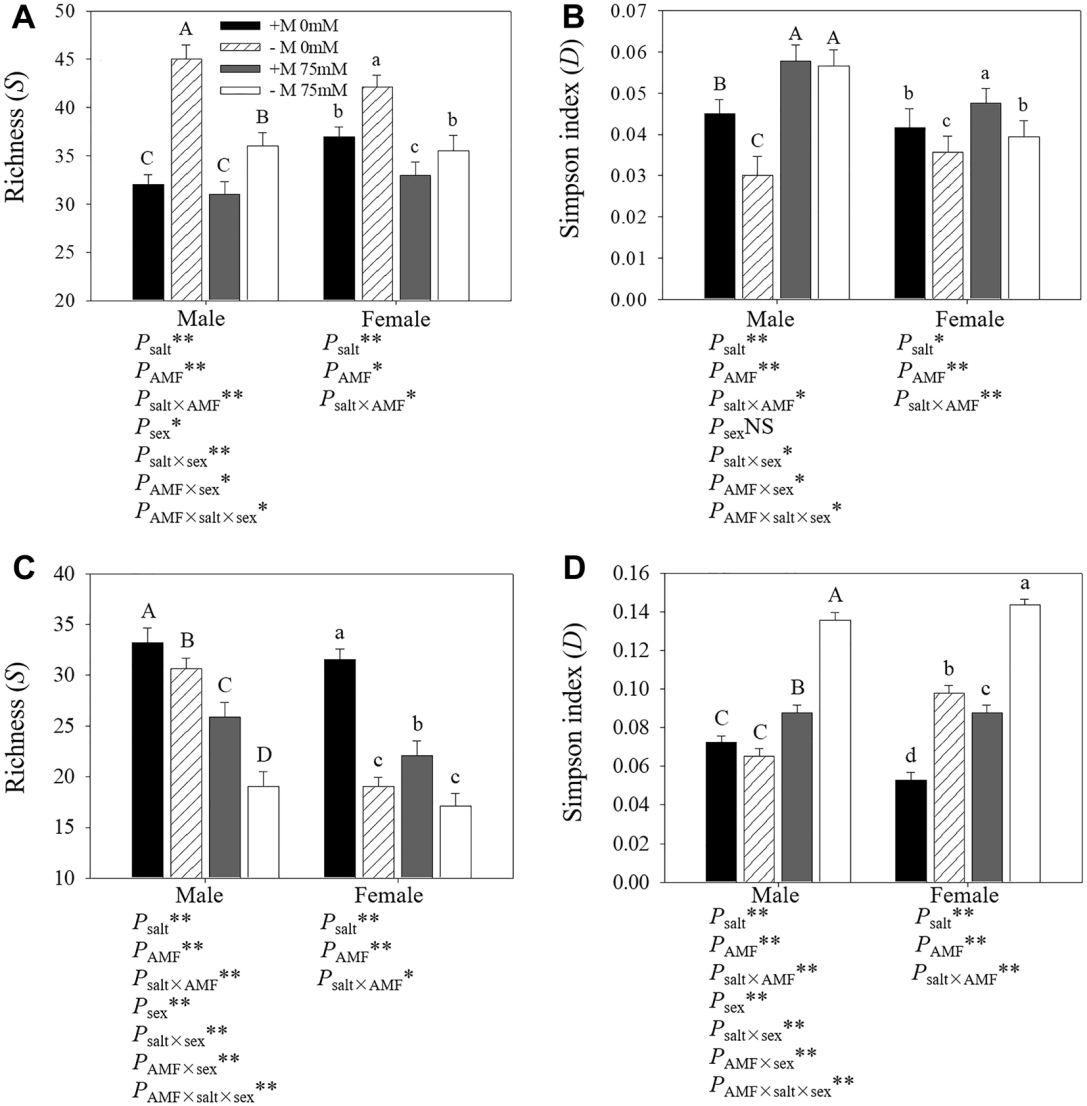
Influence of exogenous AM inoculation and salt stress on bacterial (**A**,**B**) and fungal (**C**,**D**) community in the rhizosphere of *P*. *cathayana*. +M: exogenous AM inoculation; −M: non-exogenous AM inoculation; 0 mM: without salt stress; 75 mM: under salt stress; **: significant effect at *P* ≤ 0.01; *: significant effect at 0.01 ≤ *P* ≤ 0.05; NS: no significant effect *P* > 0.05. Different letters (capital letters for males and lowercases for females) indicate significant difference (*P* ≤ 0.05), the data are means ± SD (n = 6).

Based on the digital brightness and location results of bacterial and fungal DGGE bands (Fig. 1, figures of full-length blots were supplied in Supplementary Figs. 1 and 2), we obtained the diversity indices for microbial community structure (Fig. 3). Salt stress limited fungal *S* in rhizosphere of females from both inoculation treatments, and males with exogenous AM inoculation treatment. Furthermore, salt stress stimulated *D* of fungi, while exogenous AM inoculation treatment went against the effect of salt. Among bacteria community, contrary to fungal responses, exogenous AM inoculation treatment stimulated bacterial *S* significantly of soil from both genders, while salt stress showed negative effect except in females without exogenous AM inoculation. For bacterial *S* of soil from females, exogenous AM inoculation treatment showed very significant positive effect, especially under salt conditions of 0 mM. Contrary to results of bacterial *S*, salt stress induced *D* of bacteria. However, exogenous AM inoculation treatment showed a positive effect on bacterial *D*, except male seedlings under 75 mM salt conditions.

Two-way and three-way ANOVA results suggested that except gender effect on bacterial *D*, effects of salt treatment, AM inoculation treatment, gender treatment and interactions of any 2 factors and all 3 factors were significant on bacterial and fungal *S* and *D*.

### Correlation analysis between environmental factors and microbial species

**Figure 4:**
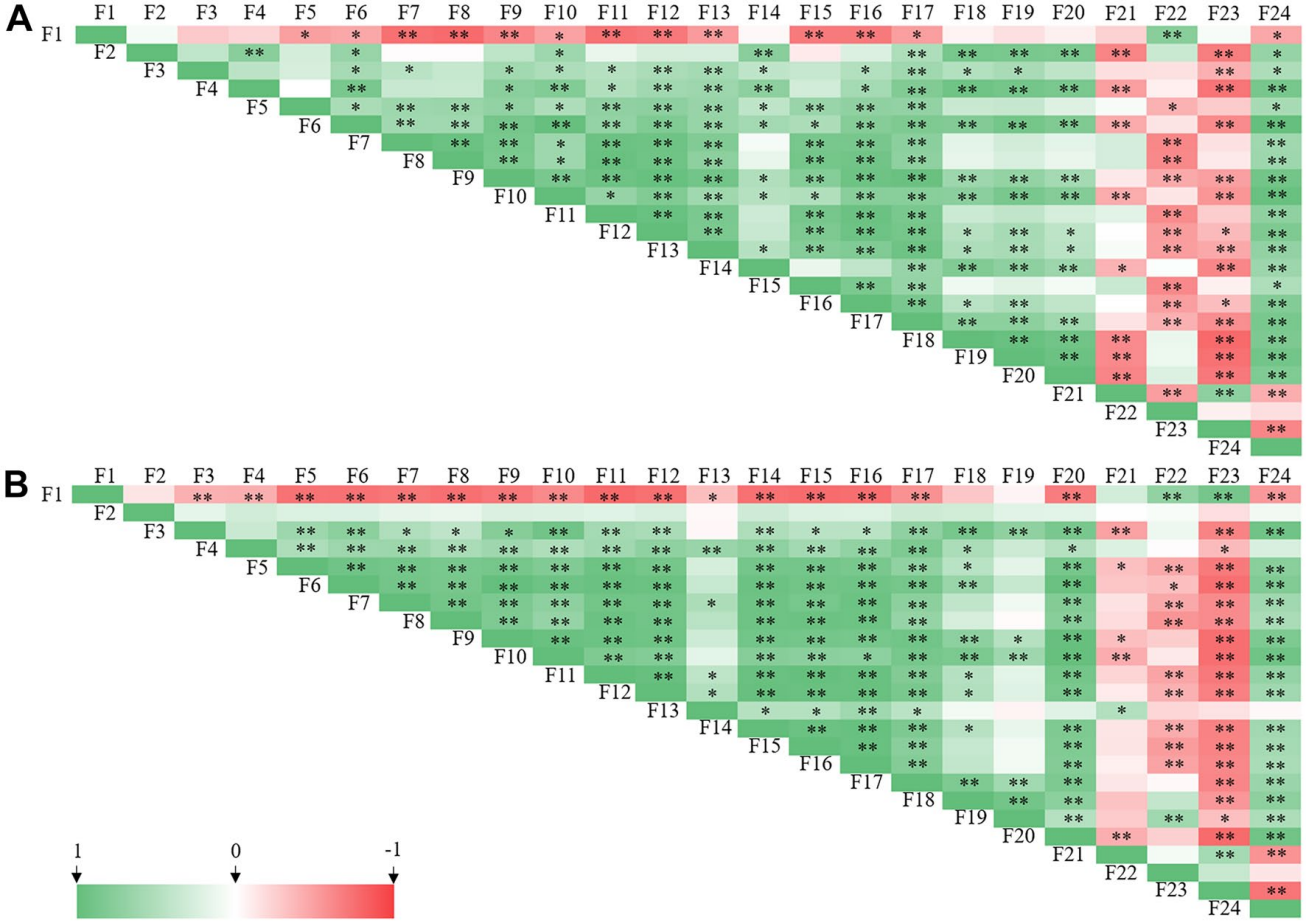
Correlation analysis between soil properties, plant growth parameters, AM status and microbial attributes of male (**A**) and female (**B**) seedlings. *Note*: F1: EC; F2: SOC content; F3: available N; F4: available P; F5: available K; F6: ALP activity; F7: surcease activity; F8: urease activity; F9: hydrogenase activity; F10: catalase activity; F11: GRH; F12: biomass accumulation; F13: N content of the leaf; F14: N content of the root; F15: P content of the leaf; F16: P content of the root; F17: TG content; F18: EEG content; F19: AM colonization rate; F20: AM spore density; F21: Richness of bacteria; F22: Simpson’s diversity index of bacteria; F23: Richness of fungi; F24: Simpson’s diversity index of fungi.

The correlation analysis suggested that significant correlations existed between plant growth parameters, microbial community attributes and soil properties (Fig. 4). Some differences existed between correlation results of male and female seedlings. Regardless of gender, soil properties (from F1 to F10) showed close relationship with each other, in which no significant correlation was detected between SOC and our indexes detected in female seedlings. Soil EC value had significant negative correlation with most soil properties and plant growth parameters (from F11 to F16).

Among male seedlings, plant growth parameters formed positive correlations with soil properties (except EC and SOC) and AM attributes (from F17 to F20) well, and negative correlations with bacterial and fungal richness and bacterial Simpson index, whereas all the soil properties, growth parameters and AM attributes formed significantly positive correlations with fungal Simpson index. Among female seedlings, growth parameters (except N content of the leaf) had a significantly positive correlation with soil properties (except EC and SOC), TG content and AM spore density. N content of the leaf only formed negative correlation with soil EC, and positive correlations with soil available P content, surcease activity, TG content and bacterial richness. Fungal richness and Simpson index showed contrary correlations with other parameters: fungal richness formed significantly negative relationship with most indexes, compared with positive correlations between fungal Simpson diversity and most indexes.

### SEMs for seedling biomass and microbial community attributes

**Figure 5:**
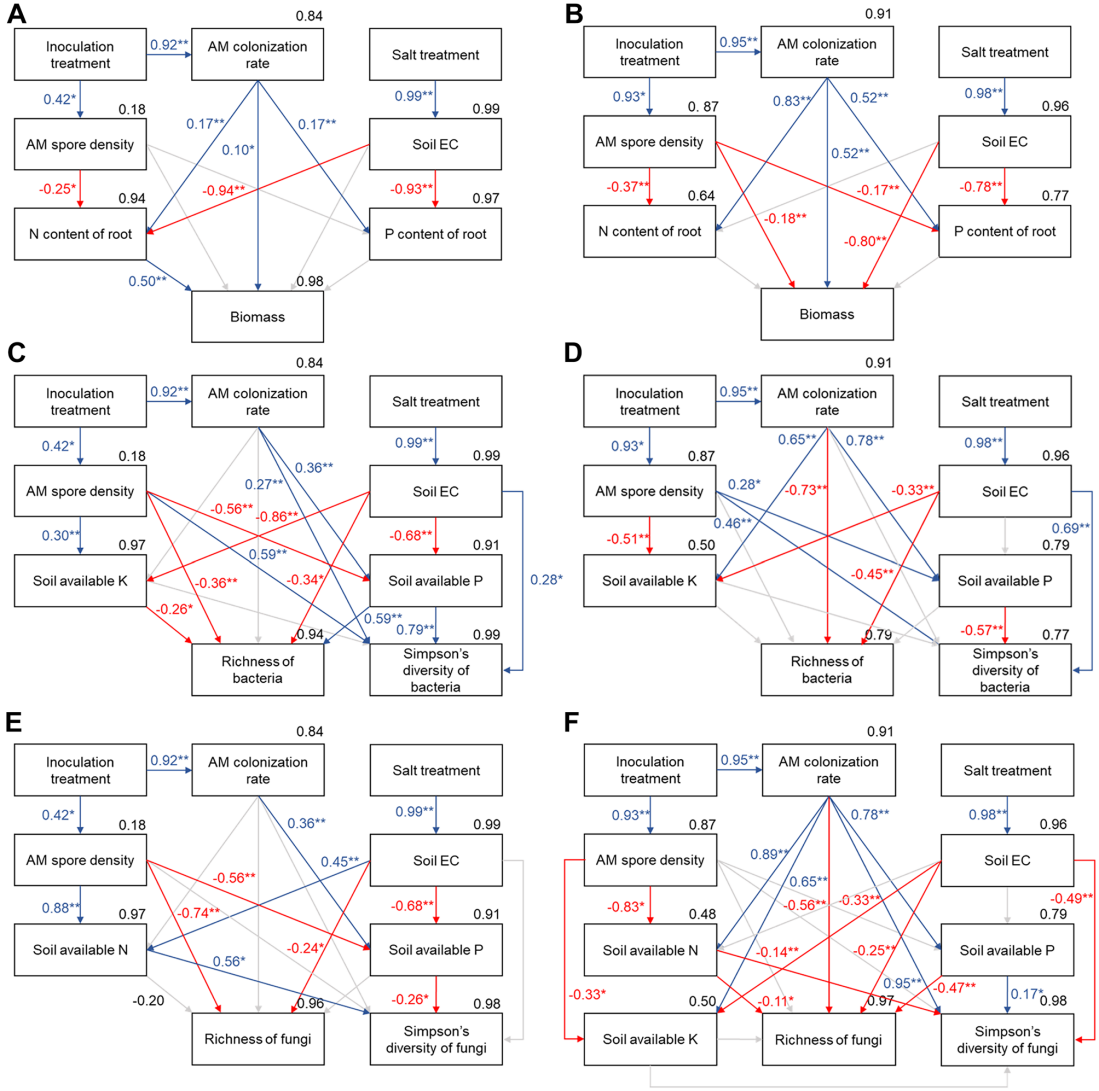
Structural equation model of casual relationships based on the result of Mantel statistics. **A**,**B** Model for biomass accumulation data of female and male seedlings; **C**,**D** Model for bacterial richness and Simpson diversity index data of female and male seedlings; **E**,**F** Model for fungal richness and Simpson diversity index data of female and male seedlings. *Note*: The number in the top right corner of each of the soil or microbial parameter boxes is the squared-multiple correlation, and the number on each line between these parameter boxes is the standardized regression-weight (*: significant at *P* < 0.05; **: significant at *P* < 0.01). Red solid lines indicate that the standardized regression weights are negative, blue solid lines indicate that the standardized regression weights are positive, and gray dashed lines indicate that the standardized regression weights are not significant (*P* ≥ 0.05) and thus their statistics are not shown.

AM inoculation treatment had a significant positive effect on AM colonization rate and AM spore density in root and rhizospheric soil of both male and female seedlings. And salt stress treatment had a significant positive effect on rhizospheric soil EC of both genders. AM colonization rate significantly positively affected the biomass accumulation of biomass of both male (Fig. 5B) and female (Fig. 5A) seedlings. For female seedlings, N content of the root had a significant positive effect on biomass, and soil EC and AM spore density had negative effects on biomass of male seedlings.

Among bacterial community attributes of both genders, richness and Simpson’s diversity index were complicatedly affected. For female seedlings, richness of bacteria was significantly negatively affected by AM spore density, soil EC and soil available K content, and positively affected by soil available P; Simpson’s diversity index of bacteria positively related to AM spore density, AM colonization and soil available P (Fig. 5C). For male seedlings, AM colonization rate, soil EC significantly affected richness of bacteria, and only AM spore density positively affected Simpson’s diversity index significantly (Fig. 5D).

Among fungal community attributes, male and female seedlings differed in contribution of parameters in affecting richness and Simpson’s diversity index of fungi. For female seedlings, richness of fungi was like that of bacteria, which was negatively affected by AM spore density and soil EC; Simpson’s diversity index of fungi was positively related to soil available N content and negatively related to soil available P content (Fig. 5E). Fungi richness from rhizosphere of male seedlings negatively related to soil available P and N contents, AM colonization rate and soil EC (Fig. 5F). Fungal Simpson’s diversity index was positively affected by AM colonization rate and negatively affected by soil available N.

## Discussion

The early response of plants to salt stress typically manifests as a reduction in the growth rates of height, biomass, and nutrient contents^15,16^, which supported our findings that salt stress caused the large reduction in plant growth parameters of both genders. SEM results revealed causal relationships between these variables in male and female seedlings. SEM results suggested exogenous AM inoculation treatment had a positive effect on AM inoculation rates in both genders, which positively affected biomass accumulation, which suggesting the helpful role of exogenous AM inoculation treatment in biomass accumulation. AM formation can promote plant growth by enhancing nutrient absorption and modifying the microenvironment in the rhizosphere of host plants^4^. The extraradical hyphal network of AM fungi allows host plants to access a greater volume of soil^17^. The secretion of mycelia can improve soil physical–chemical properties^18^. In addition, beneficial soil microbes can also improve plant tolerance to environmental stresses in direct or indirect ways^19^. Besides, different to females, biomass accumulation of males was negatively affected by soil EC. This difference may explain the gender-related differences in soil parameters and microbial community.

The soil environment is quite complex, and physical–chemical properties have complicated effects on soil microbial community^20^. The interactions between bacteria and fungi formed a dynamic balance in the rhizosphere, maintaining the physiological function of the host plant. Exogenous AM inoculation can alter the indigenous microbial community structure due to the root secretion composition change^21^, resulting in upsetting the original microbial balance. Focus on dioecious cuttings, one of the objectives of this study was to investigate different variation in the rhizosphere microbial biodiversity induced by exogenous AM fungi strains and salt. In our experiment, AM inoculation caused obvious changes in diversity index of the microbial community (especially that of the fungal community), suggesting that AM could inhibit or stimulat the proliferation of certain microorganisms to some extent^22^. Exogenous AM inoculation treatment had a greater effect on fungal community than salt treatment, whereas contrary effects were detected in the bacterial community. *R. irregularis*, a model strain of AM fungi species, is widely distributed and well adapted to various abiotic stresses, especially high-salinity soil conditions^23–25^.

AM inoculation, as well as salt stress had complex effects on the microbial community. Salt treatment, reflected in high soil EC, showed a significantly negative effect on microbial richness, regardless of gender^25–27^. Soil EC positively affected bacterial Simpson’s diversity index of both genders, but only negatively affected fungal Simpson’s diversity index of male seedlings. Bharti et al.^28^ investigated the effect of AM inoculation on the indigenous microbial community, and suggested AM inoculation had a significant effect on the microbial community. This agrees with our findings that AM spore density and AM colonization rate negatively affected microbial community attributes of females and males respectively, suggesting gender-related differences in effect of AM inoculation on microbial community^26,29^. However, the effects of treatments on Simpson’s diversity index were complicated.

As reported in this study, regardless of gender, exogenous AM inoculation treatment, reflected by increased AM colonization rate, positively affected P content of the root, as well as soil available P content. AM fungi facilitated the uptake of limited soil nutrients, especially P and N, by hosts in exchange for photosynthetic products^25^.

AM fungi can interact with other microorganisms to form soil micro-ecosystems in the rhizosphere of host plants. In our study, the formation of AM symbiosis changed certain physical–chemical properties of the rhizosphere soil, which differed between host genders^29^. Many studies have shown sex-specific response mechanisms under various kinds of abiotic stresses^30^, which may have resulted in the sex-specific microbial environments of *P. cathayana*. Thus, the sex-specific effects of AM on the microbial community of dioecious *P. cathayana* may be due to the sex-specific root exudate composition, root environment, and colonization status^31–33^. This, in turn, affects the growth and colonization patterns of soil microorganisms in different ways for the different sexes. The exogenous AM inoculation thus interacted with natural mycorrhizosphere, and affected soil properties. Sex differences could induce soil heterogeneity^34^. The similarity of target fungi in the rhizosphere of male cuttings was higher than that of female cuttings regardless of exogenous AM inoculation. When considered together with the plant parameters, these data suggest that this phenomenon may contribute to the strong adaptability of male cuttings to abiotic stresses.

However, the application of several microbial species can compensate for the above negative consequences^35^. To explore the potential sex imbalance factors in dioecious plants, the effects on soil and microbial communities in underground systems were tested. The results showed that there was a significant interaction among salt stress, exogenous AM inoculation, sex, and microbial community structure. AM inoculation significantly improved soil characteristics, changed the fungal community structure and then enhanced the growth of *P. cathayana*, which was important for easing sex imbalances and maintaining ecological stability.

## Conclusion

Salt stress has a negative effect on plant growth parameters and soil properties in the rhizosphere of *P. cathayana*, and exogenous AM inoculation can alleviate these negative effects. SEM suggested that AM inoculation, as well as salt stress had complex effects on soil properties, which affected the microbial community. By comparing the microbial community in the rhizosphere of the dioecious plant—*P. cathayana* with exogenous AM inoculation under salt stress, we aimed to provide a theoretical foundation for easing the sex imbalance status of dioecious plants under abiotic stresses through microbial bioremediation.

## Materials and methods

### Plant and soil treatment

This study involving the use of plants complies with local and national regulations. We obtained permission to collect *P. cathayana* cuttings from a poplar nursery in Yangling, Shaanxi province, China. Cuttings of 18 cm in length and 1.2 cm in diameter were collected from 120 different trees (120 genotypes: 60 of each sex) that were sampled from 15 populations (eight adult trees at the same age stage per population). The soil was collected from the upper layer (5–20 cm depth) of the poplar nursery and was sieved through a 2 mm sieve to provide the substrate. The topsoil physicochemical properties were as follows: soil organic carbon, 8.37 g kg^−1^; available K, 111.08 mg kg^−1^; available N, 33.36 mg kg^−1^; available P, 9.25 mg kg^−1^; and pH value (soil: water = 1:5), 8.0.

### AM inoculum

The AM inoculum, *Rhizophagus irregularis* BGC BJ09, provided by the Beijing Academy of Agriculture and Forestry Science, consisted of AM spores (spore density: approximately 50 g^−1^), mycelium, root fragments and soil, was propagated by *Trifolium repens* with sterile river sand. The genome of *R*. *irregularis* has already been sequenced, and it is a model strain of arbuscular mycorrhizal fungi^36^.

### Experimental design

All the cuttings were planted in 4.5 L plastic pots filled with 4 kg matrix substrate in a greenhouse. At the beginning of the experiment, holes of 1.5–2 cm in diameter and 12 cm in depth were dug in the middle of the pot. The AM inoculum was added through the hole, and the cuttings were placed into the inoculum. This experiment had a randomized block design with three types of treatment: (1) sex: male and female cuttings, (2) inoculation: (i) plants without exogenous AM inoculation (−M), (ii) plants inoculated with exogenous *R*. *irregularis* inoculation (+M), and 3) salt stress: 0 mM and 75 mM NaCl. We used 15 replications for each treatment, totaling 120 pots (2 × 2 × 2 × 15). All cuttings were planted in pots (23 cm × 13 cm) filled with soil substrate (4 kg) and grown in the greenhouse (25–30 °C, 12 h light per day). Thirty males and 30 females were inoculated with AM inoculum (20 g per cuttings). The other cuttings were inoculated with an autoclaved inoculum (20 g) plus an inoculum washing solution (10 ml), which had been filtered through a 1-μm nylon mesh to remove live inoculum. All the pots were well cared-for in the first 60 days in order to form a stable microbial community structure. After 60 days, the 30 females and 30 males that received AM inoculation were divided into two groups, each including 15 individuals. The 15 inoculated males and the 15 inoculated females were subjected to salt stress. The salt-stress group had 5 mM salt solution (200 mL) added every 2 days, 15 times, to reach the final concentration. The salt stress lasted for 2 months. The control group was treated with the same volume of sterilized water.

### Plant parameters

To record the plant response visually, we measured the stem length at the beginning and the end of the salt stress. The GRH was calculated as the height divided by the number of days. In addition, 6 plant samples were dried at 70 °C to constant weight for biomass determination. Then, the samples were separately ground and passed through a 100 μm mesh screen for the measurement of nutrient acquisition. N content was measured by a Kjeltec 8400 analyzer unit (FOSS-Tecator, Hoganas, Sweden)^37^. Phosphorus (P) content was measured by the vanadomolybdate method^38^.

### Sample collection

At the end of the experiment, the roots and soil samples at a depth of 5–0 cm were collected after removing the upper 5 cm of topsoil. Then, the samples from each treatment were pooled and used as one composite sample. Each composite root sample was put into centrifuge tubes filled with formaldehyde-acetic acid alcohol (FAA) for arbuscular mycorrhizal colonization (MC) analyses. The remaining samples were stored on ice and transported to the laboratory for DNA extraction and soil property analysis.

### AM colonization and spore density

Roots stored in FAA were rinsed three times in tap water, and root fragments cut into 1 cm pieces were stained^39^. Root colonization was examined under a microscope and evaluated as described by Giovannetti and Mosse^40^. The method of confirming AM colonization is to examine typical characteristics, including arbuscules, vesicles, spores and aseptate hypha. The data were recorded as the proportion of colonized root length. The AM spore density in dry soil was determined by wet sieving^41^. We counted AM spores under a stereoscopic microscope (40 × magnification) on plates with concentric grooves. The number of spores in 1 g of dry soil was regarded as the AM spore density.

### Glomalin determination

TG and EEG were measured using the method described by Wright and Upadhyaya^42^. Dried soil (5-mm sieved) was weighed (1 g) for measurement. The TG fraction was extracted by 50 mM sodium citrate solution (pH 8) and autoclaving at 121 °C for 60 min, while the EEG fraction was extracted by 20 mM sodium citrate solution (pH 7) and autoclaving at 121 °C for 30 min.

### Soil physical–chemical properties and soil enzyme activity measurements

The pH (water:soil, 2.5:1) was measured by a PHS–3B pH device^43^, and the electrical conductivity (EC) (water:soil, 5:1) was measured by an EM38–DD conductivity meter^44^. Soil organic carbon was determined by a K_2_Cr_2_O_7_ oxidation method^45^. The available phosphorous (P) was extracted with sodium bicarbonate and determined by molybdenum antimony colorimetry, while the available potassium (K) was extracted with sodium bicarbonate and determined by flame photometry^46^. The available N was measured as described by Weintraub and Schimel^47^. Soil urease, alkaline phosphatase (ALP), sucrase, catalase and dehydrogenase activities were assayed by the colorimetry method, disodium phenyl phosphate method, permanganate titration method, DNS method and TTC method, respectively^48^.

### DNA extraction and nested PCR

Soil samples maintained at − 80 °C were used for soil microbial DNA extraction. According to the manufacturer’s instructions, soil DNA was extracted using the E.Z.N.A. soil DNA kit (Omega, USA). Microbial diversity in the rhizosphere soil was investigated by a polymerase chain reaction denaturing gradient gel electrophoresis (PCR-DGGE) approach. PCR amplification of the 16S rRNA gene was performed with the universal bacterial primer set fD1 (5’AGAGTTTGATCCTGGCTCAG3’) and rP1 (5’ACGGTTACCTTGTT-ACGACTT3’)^49^ in the first round of PCR, performed with the specific bacterial primer set 534r (5’CCTACGGGAGGCAGCAG3’) and with 341f. with a GC clamp (5’CGCCCGGGGCGCGCCCCGGGCGGGGCG-GGGGCACGGGGGG3’) in the second round of PCR^50^. The yielded amplicons were approximately 1.4 kb and 190 bp, respectively. The reaction mixture (50 μl) contained 1 μl template, 1 μl each primer (10 μM), 25 μl 2 × Taq master mix (Beijing CoWin Biotech Co., Ltd) and 22 μl RNase-free water. DNA was amplified with a S1000 thermal cycler (Bio-Rad, USA) using the following procedure: initial denaturation at 94 °C for 3 min, followed by 30 cycles of 94 °C for 1 min, annealing at 55 °C for 1 min, extension at 72 °C for 1.5 min and a final extension at 72 °C for 5 min. The first round of PCR products was diluted 1/100 with ddH_2_O as the template for the second PCR. The second reaction was carried out under the following conditions: 94 °C for 3 min, 30 cycles of denaturation at 94 °C for 30 s, annealing at 55 °C for 30 s, extension at 72 °C for 30 s and a final extension at 72 °C for 5 min. The reaction mixture (25 μl) contained 1 μl template, 0.5 μl each primer (10 μM), 12.5 μl 2 × Taq master mix (Beijing CoWin Biotech Co., Ltd) and 10.5 μl RNase-free water.

PCR amplification of 18S rRNA was performed with the universal fungal primer set ITS1-F (5’CTTGGTCATTTAGAGGAAGTAA3’)^51^ and ITS4 (5’TCCTCCGCTTATTGATATGC3’) in the first round of PCR. In the second round of PCR, the primers for fungal ITS rRNA were ITS2 (5’CCTACGGGAGGCAGCAG3’)^52^ with ITS1-F with a GC clamp (5’CGCCCGCCGCGCGCGGCGGGCGGGGCGGGGGCACGGGGGGCTTGGTCATTTAGAGGAAGTAA3’)^53^. The yielded amplicons were approximately 1000 bp and 250 bp, respectively. DNA was amplified in the first reaction using the following procedure: 94 °C for 5 min, 35 cycles of 94 °C for 30 s, annealing at 55 °C for 30 s, extension at 72 °C for 2 min and a final extension at 72 °C for 5 min. The first round of PCR products was diluted 1/50 with ddH_2_O to be template for the second PCR. The second reaction was carried out under the following conditions: 94 °C for 5 min, 35 cycles of denaturation at 94 °C for 30 s, annealing at 55 °C for 30 s, extension at 72 °C for 30 s and a final extension at 72 °C for 5 min. PCR amplifications of fungal 18S rDNA genes were performed in a mixture volume as above. The PCR product yield was analyzed by agarose gel electrophoresis (1.0% (w/v) agarose, 140 V, 20 min) and Du Red staining in the presence of a DL2000 DNA marker (Takara Biotechnology, China).

### DGGE analyzes

DGGE analysis was carried out using the DCode universal mutation detection system (Bio-Rad, CA, USA). Fifty microliters of bacterial nested-PCR products were applied directly onto an 8% (w/v) polyacrylamide gel (37.5:1 acrylamide/bioacrylamide) containing a linear denaturing gradient from 40 to 60%, while the same volume of fungal nested-PCR product was applied to a linear denaturing gradient from 30 to 50%. Notably, the 100% denaturing acrylamide was identified as containing 40% formamide and 7 M urea^50^. The gel was initially run at 120 V for 10 min, and then the voltage was lowered to 80 V for 12 h in buffer (1 × TAE) at 60 °C. After staining with the new nucleic acid dye Du Red for 10 min, the gels were distained in deionized water for 10 min and digitally captured by a Gel Doc XR system (Bio-Rad, USA). The bands were analyzed by Quantity One software (Bio-Rad, USA). First, the auto frame lanes were selected, and the rolling disk size was adjusted to 5 to minimize the background value. Second, the bands were detected, and the parameters were adjusted to obtain the most reliable band pattern^54^. The Gauss model was applied to all lanes. Third, the tolerance was set to 4.00%, and the lanes with the most bands were selected by auto-matching. Finally, the other lanes were manually matched, and the peak densities of all the lanes were reported.

### Statistical analysis

Quantity One software (Bio-Rad, Hercules, CA, USA) was used for DGGE banding profile analysis. In general, DGGE can only visualize the dominant taxa, so the microbial communities mentioned in this paper refer to the dominant microbial communities^55^. The peak density data transferred from the DGGE profiles were imported into SPSS version 16.0 (Chicago, USA) to calculate diversity indices, including the Simpson’s diversity index (*D*) and richness (*S*)^56^. A Mantel test (0.05 based on 10,000 permutations) was conducted to compare dissimilarity matrices of the microbes by XL Stat 7.5 (Addinsoft, NY, USA). The plant parameters, soil physical–chemical properties, GRSP content, AM colonization and spore density data were analysed using SPSS version 16.0. Significant differences were detected by a three-way analysis of variance (ANOVA) and Duncan’s test.

Soil EC, SOC content, available N, available P, available K, enzymatic activities (urease, alkaline phosphatase, sucrase, catalase and dehydrogenase) were standardized. Seedlings GRH, biomass accumulation, N content in leaf and root, P content in leaf and root were transformed into standard variables. To evaluate the effect of AM inoculation treatment on seedlings growth and microbial community, AM colonization rate, AM spore density, TG and EEG were also standardized. To evaluate the effects of AM inoculation treatment and salt stress treatment, we set inoculated treatments (AM inoculated treatment as “1”, uninoculated treatment as “0”) and salt stress treatments (0 mM treatment as “0”, 75 mM treatment as “1”) and standardized as inoculation and slat stress treatment variables (Treatment). Based on the richness and Simpsons’ diversity index, fungal and bacterial Bray–Curtis similarity index was calculated and then transformed into distances using software XLSTAT (Addinsoft, Paris, France).

To determine the relationship between soil properties, seedling growth attributes and microbial community, all standardized data sets and microbial similarity indexes were transformed into distance matrices using Euclidean distance and dissimilarity index. According to the matrices, the Mantel test and partial Mantel tested were performed by controlling the influence of Treatment^57^.

In order to show causal relationship between the variables, structural equation modelling (SEM) was performed using AMOS version 22.0 (Amos Development Corporation, Meadville, PA, USA) based on results of Mantel tests^58,59^. We performed maximum likelihood solution procedures. And to evaluate model adequacy χ^2^ goodness of fit measures were used. For obvious model-data discrepancies determination, residuals and modification indices were also examined. The SEMs were computed using Inoculation treatment, Salt treatment, AM colonization rate, AM spore density, soil EC, N and P contents in root for seedling biomass; Inoculation treatment, Salt treatment, AM colonization rate, AM spore density, soil EC, available K and P contents for bacterial community attributes; and Inoculation treatment, Salt treatment, AM colonization rate, AM spore density, soil EC, available K, N and P contents for fungal community attributes.

## Supplementary Information

Supplementary Figure 1.

Supplementary Figure 2.
