# Supplementary figures and images for: Impact of salt and exogenous AM inoculation on indigenous microbial community structure in the rhizosphere of dioecious plant, Populus cathayana
Source: Sci Rep. 2021 Sep 15;11:18403. doi: 10.1038/s41598-021-97674-w (PMC8443550; doi:10.1038/s41598-021-97674-w)

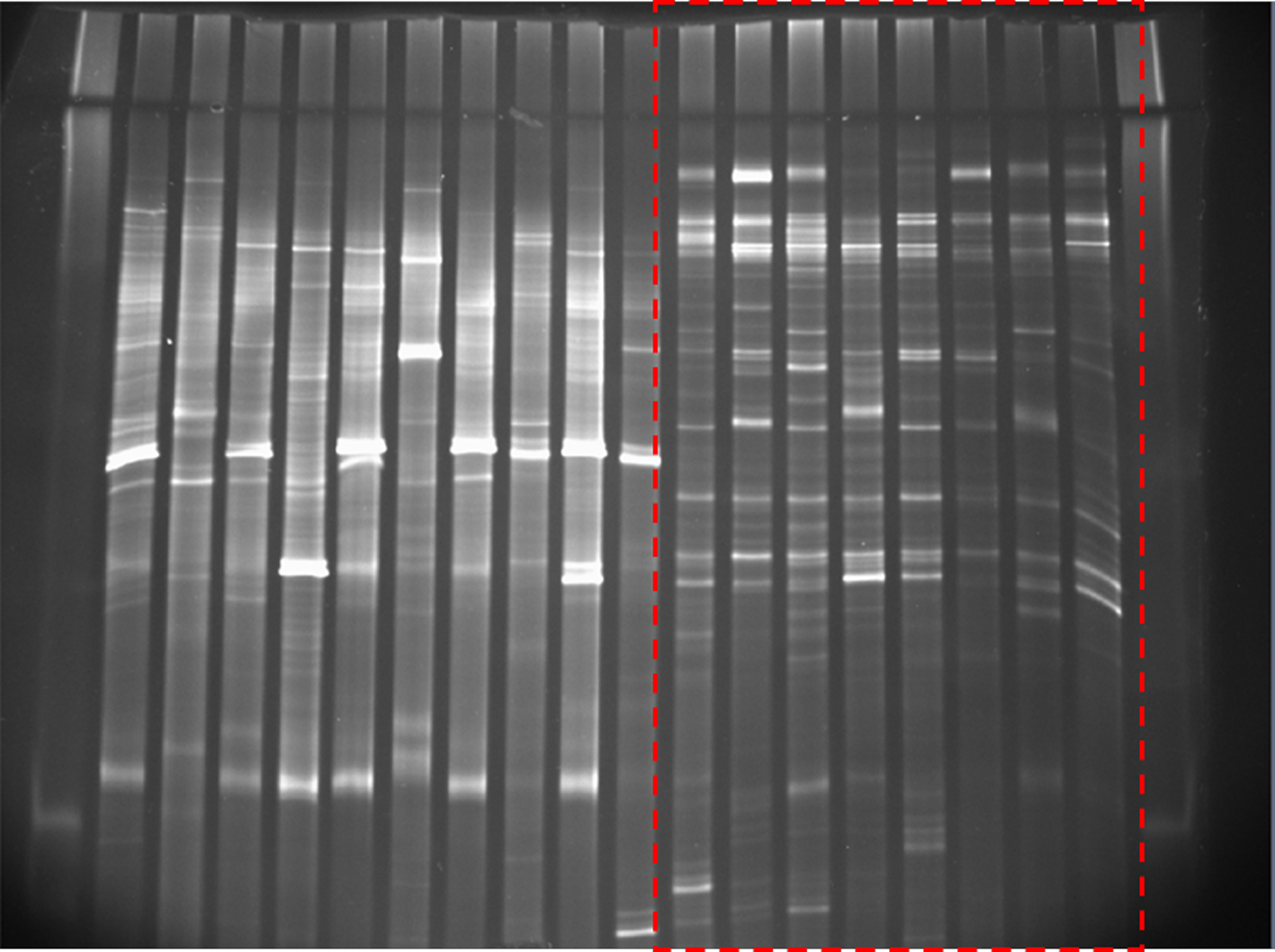

Supplement: Supplementary file 1 — Supplementary Figure 1. [file 41598_2021_97674_MOESM1_ESM.tif]

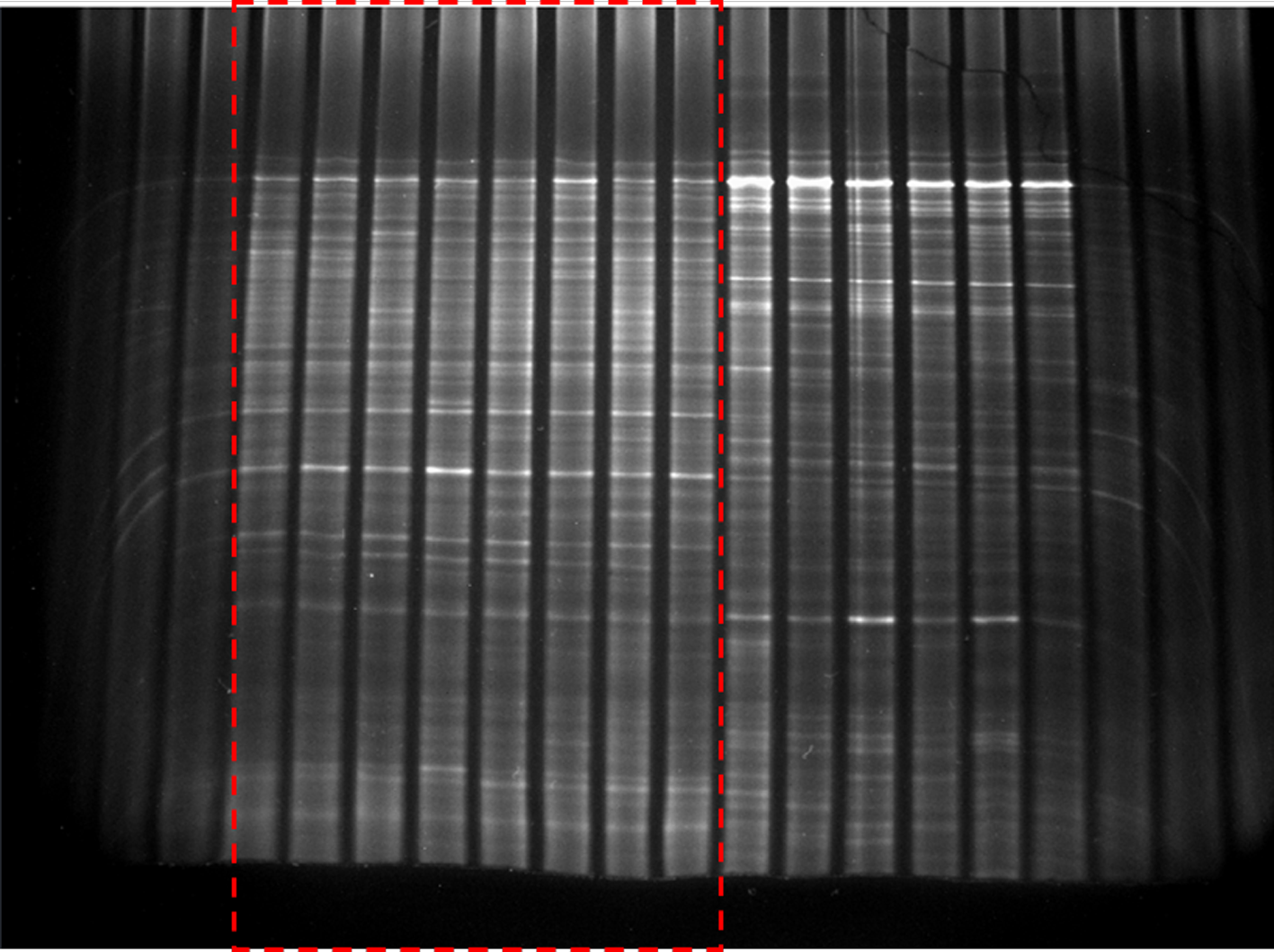

Supplement: Supplementary file 2 — Supplementary Figure 2. [file 41598_2021_97674_MOESM2_ESM.tif]
